# Anthropogenic effects on the body size of two neotropical orchid bees

**DOI:** 10.1186/s12862-022-02048-z

**Published:** 2022-08-02

**Authors:** Johannes Garlin, Panagiotis Theodorou, Elisa Kathe, José Javier G. Quezada-Euán, Robert J. Paxton, Antonella Soro

**Affiliations:** 1 General Zoology, Institute for Biology, Martin Luther University Halle-Wittenberg, Hoher Weg 8, 06120 Halle (Saale), Germany; 2 German Centre for Integrative Biodiversity Research (iDiv) Halle-Jena-Leipzig, Puschstrasse 4, 04103 Leipzig, Germany; 3 Departamento de Apicultura Tropical, Universidad Autónoma de Yucatan, Merida, Mexico

**Keywords:** Life history, Urbanization, Agricultural intensification, *Euglossa*

## Abstract

To accommodate an ever-increasing human population, agriculture is rapidly intensifying at the expense of natural habitat, with negative and widely reported effects on biodiversity in general and on wild bee abundance and diversity in particular. Cities are similarly increasing in area, though the impact of urbanisation on wild bees is more equivocal and potentially positive in northern temperate regions. Yet agriculture and urbanisation both lead to the loss and alteration of natural habitat, its fragmentation, a potential reduction in floral availability, and warmer temperatures, factors thought to be drivers of wild bee decline. They have also been shown to be factors to which wild bee populations respond through morphological change. Body size is one such trait that, because of its relation to individual fitness, has received growing attention as a morphological feature that responds to human induced modification in land use. Here, we investigated the change in body size of two sympatric orchid bee species on the Yucatan Peninsula of Mexico in response to urbanization and agricultural intensification. By measuring 540 male individuals sampled from overall 24 sites, we found that *Euglossa dilemma* and *Euglossa viridissima* were on average smaller in urban and agricultural habitats than in natural ones. We discuss the potential role of reduced availability of resources in driving the observed body size shifts. Agricultural and urban land management in tropical regions might benefit wild bees if it encompassed the planting of flowering herbs and trees to enhance their conservation.

**Supplementary Information:**

The online version contains supplementary material available at 10.1186/s12862-022-02048-z.

## Introduction

The human population has been projected to reach 10 billion by 2050 [1], with the proportion of the human population living in urban areas also likely to rise at a very high rate such that urban land cover is forecast to reach 0.6–1.3 million km^2^ by 2050, an expansion of 78–171% of the global urban land area of 2015 [2]. Urban development leads to habitat loss and degradation, to a high density of impervious surfaces such as roads, to pollution of the air, soil and water, and to considerable light and noise pollution [3, 4]. For these reasons, the urban ecosystem is generally considered inhospitable for wild fauna [5], though recent studies in northern temperate cites have suggested that wild bees may benefit from the supply of flowers and nesting sites in gardens and public parks [6–8].

To support an ever increasing urban population, the area needed to grow food has increased, with a major impact on the biosphere [9], at the cost of millions of acres of tropical forests [10]. Given this degree of loss, it should not come as a surprise that insects are declining, both in species numbers and abundance [1, 11], wild bee species included [12].

Habitat loss, degradation and fragmentation are among the most important threats to wild bee abundance and species richness, primarily due to the loss of floral and nesting resources that fragmentation and habitat loss entail [13]. Furthermore, malnutrition, pesticides, pollutants, pathogens and competition with managed bees have all been linked to bee decline [14]. Interestingly, this is even more apparent for agriculture landscapes than for cities as, in the context of modern, industrial agriculture, rural areas can be completely deprived of any patch that might provide some resources to wild bees while viable habitats, albeit necessarily fragmented [15] and potentially small and isolated, can be widespread in cities in the form of parks, community and private gardens [7], at least in northern temperate regions.

Human induced climate change is another, widely perceived driver of bee decline, especially in relation to the rising temperatures that it causes, leading to species migration towards cooler areas at higher latitudes and altitudes [16]. Cities can be even warmer than surrounding rural areas because of the urban heat island (UHI) effect [17], which itself can alter species richness and abundance [18].

In response to these environmental stressors, species are not only migrating [16], they are also changing, both physiologically [19, 20] and morphologically, particularly in body size [21]. Alteration in body size is not surprising given that it is one of the most fundamental life-history traits with pervasive effects on individual fitness [22, 23]. Body size affects important life-history attributes such as fecundity and longevity [24, 25]. It also correlates with energetic expenditure, diet, thermoregulation and home range size [26]. In bees, body size influences foraging range [27], genetic differentiation [28] and pollination efficacy [29].

Because of these fitness implications, body size is expected to respond to stressors associated with human activities such as increased fragmentation, increased temperatures [21], and decreased availability of resources [30]. Fragmentation is expected to favour larger bees as they are better suited to overcome distances that might separate green patches with floral and nesting resources [31]. Higher temperatures are expected to favour smaller bee individuals of a species and also smaller species because of the higher metabolic costs they impose on larger organisms [23, 32]. As the quantity and quality of food received during larval stages determine the final adult size [23, 33, 34] and larger bees likely require more resources to successfully raise their brood [35], reduced resource availability is also expected to be associated with smaller body sizes. Larger bee species have indeed been found to be more vulnerable to the resource depletion typical of human affected habitats [30, 36–38]. Given this set of considerations, expectations as to how intraspecific body size might change in human impacted areas are mixed: highly fragmented urban landscape are expected to favour bigger bees. But, because of the urban heat island effect [17], cities are also warmer, which leads to the prediction that smaller bee body size is favoured in cities compared to surrounding areas. Similarly, sites with intensive agriculture might also be relatively poor in floral resources, especially if monocultures consist of unsuitable plants with limited availability of pollen and nectar [39], and thereby favour small body size.

Some of these predictions have received partial support; bees have been found to be larger in the fragmented urban landscape [29], and a positive association has been shown between landscape fragmentation and body size [40]. In contrast to these results, Eggenberger et al. [41] found that urban bumble bees were smaller compared to conspecifics in rural populations, interpreted as an indication for temperature and floral availability to be more important in determining intraspecific body size compared to the advantage of being large in fragmented areas [41]. The inconsistency of results across studies suggests that the response of intraspecific body size to anthropogenic challenges may be site- or species-dependent.

These equivocal results highlight the need for additional investigation into body size as a response trait. Most studies on body size responses to anthropogenic change have to date been carried out in temperate regions, often with bumble bees as model organisms (but see [42]). Results from studies undertaken on tropical bees are badly needed [43] because tropical habitats are among the most fragile [44] and the rate of deforestation they currently undergo is higher than in any other part of the world [10, 45]. Latin America is one of the regions in the world with the highest rate of urban growth, including in the Yucatan Peninsula of Mexico [46]. Though the Yucatan Peninsula holds the largest tropical forest biosphere in Mexico, since mechanised agriculture began in the 1960s, 10% of the region’s forests have been disturbed by anthropogenic activities, with cascading effects on habitat and biodiversity loss [45]. Moreover, it is now unequivocal that rapid urbanization, especially because of its effects on vegetation cover, has an impact on the local climate [47, 48] through the urban heat island (UHI) effect [49, 50]. Data collected for the Yucatan Peninsula’s main city of Merida, the best studied city in Yucatan [46, 51], have confirmed this pattern: Merida has experienced a land surface temperature increase of at least 3 °C in the last two decades [46] that may be attributed to a conspicuous contraction in vegetation cover [46]. Because of these dramatic and quick developments, tropical cities often differ markedly from those in temperate zones: temperate zone cities tend to be rich in parks and gardens and therefore relatively generous in their offer of floral resources for bees [7, 52, 53], while cities in tropical zones tend to be uninterrupted cement and asphalt expanses with very few green patches and therefore relatively poor in floral resources [42, 54].

*Euglossa viridissima* and *Euglossa dilemma* are two cryptic, sympatric orchid bee species (tribe Euglossini) distributed from Costa Rica to Mexico, with *E. dilemma* having recently extended its distribution to subtropical Florida [55, 56]. These two medium-sized (∼ 12 mm in length) metallic green orchid bees are frequently encountered within cites of their Neotropical distributional range, suggesting they are well adapted, more so than other *Euglossini* bees, to hotter and drier environments and seem to be less dependent on intact tropical forest. Importantly for this study, they are abundant in suburban parks and gardens or heavily degraded dry forests [55]. Pollen resources of these sibling species overlap substantially, with little or no resource partitioning [57]. As for other orchid bees, they are also long-distance pollinators with considerable flight capabilities and use environmental odours for intraspecific communication, facilitating the capture of males [58–60]. Both *E. viridissima* and *E. dilemma* have been described as primitively eusocial [61–63].

Here we compare male body size of *E. viridissima* and *E. dilemma* that were collected on the Yucatan Peninsula (Mexico) from sites that differed in land use and level of anthropogenic disturbance to investigate the question of whether stressors related to anthropogenic activities, namely urbanization and agriculture intensification, have an impact on intraspecific body size. We formulated the following predictions: (1) if habitat fragmentation were the main driver of variation in body size, as in many temperate bumble bees, then these two orchid bees would be larger in more fragmented urban or agricultural habitats; (2) if temperature were the main driver of body size, then body size would be smaller in urban sites; (3) lack of resource supply should negatively affect bee body size in urban and agricultural sites. We further assess how body size of individuals from islands compare with body size of individuals from locations on the mainland. Islands are particular in that they are more isolated than any suitable fragment within the heterogeneous matrix of the mainland landscape and notoriously poorer in resources. On islands we would therefore expect larger sizes if isolation were to sort for more vagile individuals migrating from the mainland, but smaller individuals if body size were to reflect a paucity of resources.

## Results

In total, 540 male individuals of the two species *E. dilemma* and *E. viridissima* were individually measured, 140 from urban sites (*E. dilemma*, n = 100; *E. viridissima*, n = 40), 180 from agricultural sites (E. dilemma, n = 160; E. viridissima, n = 20), 180 from natural sites (*E. dilemma*, n = 140*; E. viridissima*, n = 40) and 40 from islands (*E. dilemma*, n = 40; *E. viridissima*, n = 0). Body size ranged from 3.06 to 3.87 mm for *E. dilemma* (n = 440, X̅ = 3.47 ± 0.14 SD) and from 3.21 to 3.91 mm for *E. viridissima* (n = 100, X̅ = 3.54 ± 0.13 SD).

When body size was analysed using the two species data sets together, we found a habitat effect (LMM: χ^2^ = 11.33, df = 3, p = 0.01), but also a species effect (LMM: χ^2^ = 9.39, df = 1, p = 0.002), with *E. viridissima* males being on average 2.2% bigger than *E. dilemma* males. Nevertheless, we did not find evidence for an interaction between habitat type and species identity on body size (LMM: χ^2^ = 2.18; df = 3, p = 0.53), suggesting that the two species respond similarly to the different types of habitat. Tukey’s HSD post hoc test showed that *Euglossa* males in natural habitats were significantly bigger in size than in all other habitat types, while no significant difference in size was found among agricultural, city or island habitat types (Additional file 1: Table S1).

**Fig. 1:**
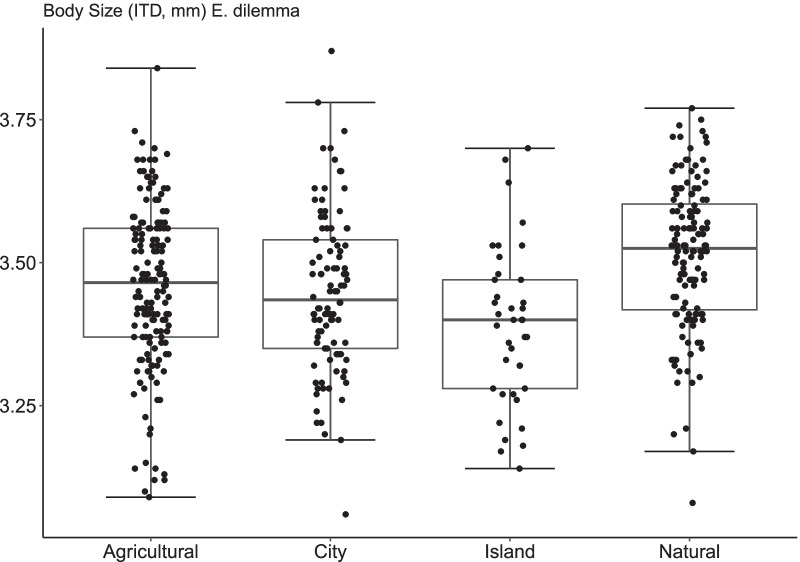
Medians and interquartiles of body size (measured as intertegular distance, ITD) for *Euglossa dilemma* across four the four habitats ‘Agricultural’, ‘City’, ‘Island’ and ‘Natural’

A significant effect of habitat on male body size was also found when *E. dilemma* was analysed alone (χ^2^ = 11.19, df = 3, p = 0.01). Tukey’s HSD post hoc test showed that *E. dilemma* males in natural habitats were significantly bigger in size than in all other habitat types, while no significant difference in size was found among agricultural, city or island habitat types (Fig. 1, Additional file 7: Fig. S1, Additional file 1: Table S1).

**Fig. 2:**
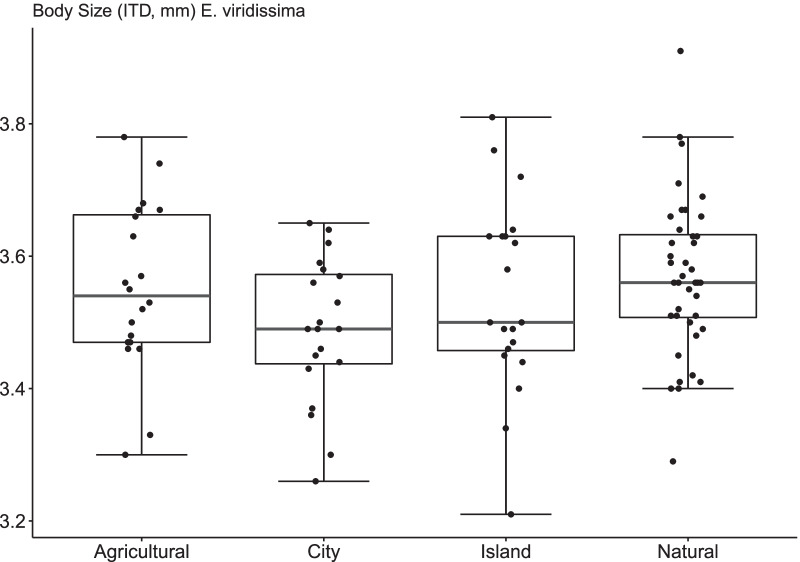
Medians and interquartiles of body size (measured as intertegular distance, ITD) for *Euglossa viridissima* across four the four habitats ‘Agricultural’, ‘City’, ‘Island’ and ‘Natural’

We could not confirm a significant ‘habitat’ effect on male body size in *E. viridissima* (χ^2^ = 5.73, df = 3, p = 0.13), but direct pairwise comparisons revealed that individuals sampled in the natural (N) habitat were on average significantly bigger than individuals sampled in cities (C) (Fig. 2, Additional file 8: Fig. S2, Tukey HSD; Z = 2.37, p = 0.02, Additional file 1: Table S1). There was no effect of habitat type on the coefficient of variation of body size, either for *E. dilemma* (F-statistic: 0.60 on 3 and 18 DF, p-value: 0.63), or for *E. viridissima* (F-statistic: 0.26, 3 and 1 DF, p-value: 0.86).

Land use variables were assessed at n = 24 sampling sites, including n = 6 urban sites, n = 9 agricultural sites, n = 9 natural sites and n = 3 islands. The proportions of the different land use variables investigated differed among the four habitat types, apart from ‘water’ and ‘seminatural areas’ (Additional file 2: Table S2). Apart from ‘water’, results did not change when comparison were made at the scale at which each variable correlated most with body size (Additional file 2: Table S2).

Of all the land use variables that were used as predictors for body size across sites, none was significant (Additional file 3: Table S3).

## Discussion

Our results revealed a significant shift to smaller body size of *E. dilemma* males in anthropogenic ecosystems such as cities and agricultural areas compared to natural habitats, suggesting that body size in bees is a sensitive response trait to anthropogenic changes, such as urbanization [29, 41, 42] or agriculture intensification [31, 40, 64, 65].

Our study focused on males because they are very easily captured (see Methods), while orchid bee females are extremely difficult to sample. While recognising this as a limitation of our study because size dependent fitness implications might be different between the two sexes, we note that there is very limited sexual size dimorphism between males and females [66]. Females are therefore likely to respond physiologically to environmental stressors in a similar manner as males.

We did not find *E. viridissima* males to vary significantly across habitat types, very likely because of the small dataset. Nevertheless, city males of *E. viridissima* were, like those of *E. dilemma*, on average smaller than conspecifics caught in natural areas, indicating an urbanisation effect on *E. viridissima* males, too. For *E. dilemma*, our results are based on a well replicated statistically powerful sampling design, in nine independent regions on the Yucatan Peninsula of Mexico. That results for *E. viridissima* partially confirm those for *E. dilemma* suggests that the two species likely respond similarly to human induced changes, as also indicated by the lack of interaction effect between habitat and species identity. The two species were significantly different in size, but their nutritional niches greatly overlap [57] and there are no known differences in their physiology or foraging behaviour that might explain this difference. Among the land use variables analysed, none could be singled out as determining the habitat effect we detected.

Three main drivers associated with anthropogenic disturbance that are expected to have an effect on body size in bees are: fragmentation, increased temperatures and scarcity of food resources. The increase in habitat fragmentation of anthropogenic habitats is expected to be associated with bigger body sizes, as seen in bumble bees in temperate zone cities [29], as bigger bees can likely fly the longer distances needed to reach favourable fragments. Alternatively, the increased temperatures of urban habitats and the scarcity of food resources of both urban and intensively cultivated habitats are expected to be associated with a shift to smaller body sizes [21].

Our detection of a shift in body size suggests that local conditions, also in the tropics, can have a significant and appreciable effect on male orchid bee body size. Our results exclude fragmentation, at least as an important driver of the shift in body size, because we found bees to be smaller in cites and agricultural habitats. This could partly be attributed to the fact that orchid bees are known to fly long distances [67] and therefore they may not be sensitive to increased habitat fragmentation. In the following, we discuss the possible relative roles of the other two main drivers, temperature and availability of resources, in explaining the patterns of variability we observed.

Given the generality of the UHI phenomenon [51, 68], and that urbanisation processes in the Yucatan Peninsula are similar across its cities [51], we assume that the other cities in this study are, like the well-studied Merida (see introduction), significantly warmer than their corresponding rural and natural areas. We therefore plausibly consider temperature as a potential factor behind the decrease in body size of orchid bees in the urban sites compared to the natural sites of this study.

Nevertheless, ongoing contraction and degradation of natural vegetation cover (for Merida ca. 16,000 ha between 1995 and 2014 [46]) and its replacement with artificial materials (ca. 9700 ha in Merida [46]), the main consequence of urban growth, are not only the cause of temperature increase, but also likely result in a reduction in floral resources and a change in vegetation composition [52], with detrimental effects on flower visiting insects [5], possibly due to some kind of nutritional imbalance [69]. Therefore in the cities considered in this study, the two main acknowledged potential drivers, increased temperatures and reduction of resources, are possibly affecting orchid bee body size concomitantly. City pollutants have also been shown to affect foraging efficiency [70] and pollination services [71], and might therefore be potential factors reducing body size.

Our results support the context-dependent nature of the response of body size to urbanisation. While cities of temperate zones can have a positive effect on body size ([29] but see [41]), tropical cities seem to affect body size negatively, as also seen in the stingless bee species *Nannotrigona perilampoides* in the city of Merida [42]. Tropical cities are considered less hospitable for bees [42, 54] than their temperate counterparts [7, 52, 53], mainly because of the many parks and gardens that populate the latter that are scarce in the former [72, 73].

Consistent with the possibility that lack of floral resources is a driver of body size shifts in *E. dilemma* and *E. viridissima* is our observation that bee body size in the agricultural sites was smaller than in natural sites (forest). As agricultural sites in rural areas are likely to be cooler than city sites [17], the reduction in body size we observed in agricultural sites is probably due to reduced availability of resources. Maize, one of the predominant crops in the agricultural sites, is not among the plant species on which *E. dilemma* and *E. viridissima* feed [57]. Reduced floral resources are nevertheless only one aspect of the many factors associated with habitat modifications through human intervention in agricultural sites. Other important stressors, which might all contribute to the effect on body size that we observed, might be increased pathogen load through spillover from managed honey bees [74–76] and competition with honey bees, which are present in Yucatan at high density [77]. Nevertheless, neither pathogen spillover nor competition with managed bees for floral resources have been directly studied in Yucatan.

Agricultural intensification is generally associated with landscape simplification and increased pesticide load [65, 78]. Renauld et al. [65] observed a reduction in body size associated with agricultural intensification in the ground nesting bee species *Andrena nasonii* in the USA, suggesting that landscape simplification reduces the overall quantity, quality and distribution of resources, with negative effects on offspring provisioning. Even if agriculture in Yucatan is not as intense as in Europe and USA [79], it has intensified since the beginning of the 1960s [45]. It has inexorably transitioned from the traditional slash-and-burn maize-bean-squash ‘milpa’, which was ecologically and economically sustainable as long as population pressure was low enough to allow a prolonged time for forest regeneration [80], to an intensive agricultural production for the Mexican market with higher reliance on pesticides, herbicides and fertilizers [81]. The negative effect of pesticides on body size has been documented in bees [82, 83] and might be due in part to pesticides impairing pollen foraging capabilities by affecting learning and memory [84, 85]. Loss of suitable nesting and foraging resources in intensively cultivated areas [86] are known to affect foraging times, distance and frequency of female bee provisioning trips and, therefore, indirectly, larval diet [65]. As larval development is dependent on pollen diversity and quality, their reduction will likely have a negative effect on adult bee body size [33, 87].

Despite the undisputable negative effects of lack of resources on bee body size, it remains to be discussed whether the body size shift we observed is due to the paucity of resources (in quality and quantity) in urban and agricultural habitats, which had an effect on the size or quality of the larval provision mass of the males we sampled in those locations (first possible explanation), or whether it results from a process of size re-distribution of male orchid bees in the mosaic landscape they experience, with larger bees sorting themselves into resource-rich areas (second possible explanation). Orchid bees, particularly males, have relatively large home ranges [59], can fly long distances [67] and have great dispersal capabilities, which are also the reasons why *E. dilemma* and *E. viridissima* [88], like other orchid bees [89, 90], exhibit low genetic differentiation. Floral resource limitation is a more plausible explanation for smaller body size in species with low dispersal capabilities [42]. Given that orchid bee males readily move among contrasting habitat types [91], we therefore consider ecological sorting to be a plausible explanation for the size differences we observed. We note, though, that this explanation does not exclude the other; both floral resource limitation and ecological sorting of adults could act in unison. The differences in body size we observed would then be determined by the distribution of resources across habitat types and the increased ability of larger individuals to access them, which is what is expected to happen under the predictions of the so called ‘silver spoon effect’ [92]. The positive relationship between the individual male attributes (body size) and the quality of the habitat in which we found them (higher resource availability) could be then be explained by the ‘search hypothesis’ [92], according to which the quality of the habitat in which an individual will ultimately settle depends on the trait that makes it a good searcher i.e. large body size in the context of our study. This is an intriguing possibility that deserves greater attention because understanding the mechanisms of trait-mediated species-specific responses may also explain how anthropogenic changes to the natural landscape might affect population persistence [93].

We found a trend for island bees to be smaller than those from all other habitat types, further supporting the idea that the paucity of food resources is one probable driver of shifts in body size in these neotropical bees. Genetic analyses of these bees [88] and of *Euglossa cordata* [94] have shown that island populations are genetically less diverse. Moreover even if genetic structuring is relatively modest [88, 94], it increases with isolation [94]. These findings indicate that islands population of orchid bees are relatively isolated from the mainland. Thus, the trend to smaller sizes on islands could be interpreted as reflecting the local (poorer in resources) conditions. Generally, island populations are known to differ in body size from their mainland relatives and evolve either gigantism or dwarfism, mainly according to the so-called ‘island rule’ [95, 96]. This rule has been revisited by [97] to explain body size distributions across islands of different sizes as the composite effect of limiting resource availability, which should drive body size shifts towards smaller individuals, and predation and competitive release, which should drive body size towards bigger individuals. However, most studies on the body size of insular animals have focused on vertebrates [98], with patterns of body size shifts of insular insects being scantly documented. Exceptions are two studies: Spengler et al. [99] found that body size (averaged across bees and wasps) decreased with increased island isolation; Palmer [100] found a pattern that fitted the expectations of the ‘island rule’ in a tenebrionid beetle: a bell shaped relationship between body size and island area, with reduced body size on small islands potentially due to scarcity of resources and reduced body size on large islands explainable in terms of intensified competition and predation. Palmer’s [100] results suggest that the ‘island rule’ can be extrapolated to insects, and support the plausibility of limiting resources as a possible explanation for the trend to smaller bees on islands we observed in our study. Importantly, our results on the body size of island bees point to the potential of islands as natural laboratories for a further understanding of the ecology of body size in bees.

Finally, even if patterns we observed in two orchid bees are consistent with the view that body size is governed by the availability of resources and temperature, none of the land use variables, which might be considered proxies for resources (forest, urban green areas or semi-natural areas) or for temperature (proportion of impervious surfaces) were significant predictors of body size. We can think of three possible reasons, for this negative result: (1) the resolution at which the land use variables were assessed (by visual inspection of google earth maps) was too coarse; (2) we did not measure important variables such as level of pollution, pesticide load, honey bee colony density, vegetation composition, flower abundance and richness, and temperature that might have been important explanatory variables for body size; and (3) habitats are more than the mere sum of a few land use variables and the cause of body size is too complex to be ascribed to them, without consideration of their interactions with other biotic factors such as competition or predation.

## Conclusions

As pollinators, bees are of indisputable economic and ecological value [101, 102]. This is also true for orchid bees, key pollinators of plants from approximately 30 families, including valuable crops and many species of orchids. Our results seem to point to floral resource availability as an important determinant of body size shifts in orchid bees, as the habitats in which bees in this study were found to be smaller (cities, agricultural areas and islands) are consistently poorer in resources than natural sites. Nevertheless, more accurate data on temperature, precipitation, resource availability (resin, nectar and pollen, flower abundance and richness), foraging efficiency, bee movement, pesticide use, and the intensity of pollution in tropical cities and surrounding agricultural areas would help provide a more nuanced analysis of the variables that are involved in determining body size shifts, which would allow more targeted conservation measures to maintain healthy bee populations.

## Methods

### Sampling

**Fig. 3:**
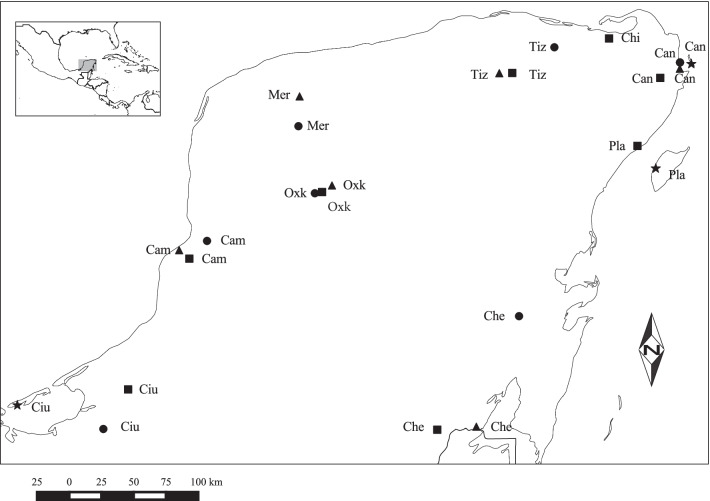
Map of Yucatan Peninsula indicating locations where males of *Euglossa dilemma* and *Euglossa viridissima* were sampled. The three letter codes refer to the regions to which sampling locations belong (See Table 1). The four different symbols correspond to the four treatments considered in this study: dots = natural; squares = agricultural; triangles = city; stars = island

*Euglossa dilemma and E. viridissima* were collected on the Yucatan Peninsula of Mexico using synthetic odour baits, a standard sampling method that exploits the tendency of orchid male bees to be attracted to the floral odours that they collect to attract females [103] (see [88] for more details on the sampling methodology). Individual male bees were sampled from 24 sampling sites across 9 different regions (Fig. 3). Each region was subclassified in four site types corresponding to different habitat types: ‘Natural’ (N) covered by at least 80% natural forest, characterised by trees at least 10 m high and with no evidence of recent human impact; ‘Agricultural’ (A), localities situated within an agricultural matrix with maximum 20% forest cover; ‘City’ (C), indicating an urban area; and ‘Island’ (I), indicating sampling on an offshore island for regions by the coast (Fig. 3 and Table 1). At each of 22 sites, ca. 60 males of *E. dilemma* were sampled from January to May 2010, totalling 1429 bees [88]. For *E. viridissima* an average of 46 males were collected at each of 5 sites from February to March 2010, totalling 257 bees [88]. The number of baited individuals per total baiting time was measured, providing an estimate of abundance per hour, which did not differ significantly between species (Mann–Whitney *U* Test: W = 1.27, p = 0.16), region (Kruskal–Wallis chi-squared = 7.89, df = 8, p-value = 0.45) or habitat type (Kruskal–Wallis chi-squared = 4.74, df = 3, p-value = 0.19).

**Table 1:** Sites at which male *Euglossa dilemma* and *Euglossa viridissima* were collected (n = 20 for each site)

Site	Region	Region Code	Habitat	Mean Body Size *E. dilemma* (mm)	Coefficient of variation *E. dilemma* (CV)	Abundance *E.dilemma* (number of bees caught per hour)	Mean Body Size *E. viridissima* (mm)	Coefficient of variation *E. viridissima* (CV)	Abundance *E.viridissima* (number of bees caught per hour)	Latitude	Longitude
Cancun_C	Cancun	Can	City	3.466	0.033	10.00	na	na	na	21°10′54.55″N	86°48′35.40″W
Cancun_N	Cancun	Can	Natural	3.544	0.016	20.29	na	na	na	21°13′43.13″N	86°48′30.16″W
Cancun_A	Cancun	Can	Agricultural	3.365	0.054	21.14	na	na	na	21° 6′48.00″N	86°57′21.74″W
Cancun_I	Cancun	Can	Island	3.401	0.031	11.00	na	na	na	21°13′2.32″N	86°43′25.23″W
Chetumal_A	Chetumal	Che	City	3.503	0.033	18.00	na	na	na	18°30′10.40″N	88°19′59.75″W
Chetumal_N	Chetumal	Che	Natural	3.465	0.046	58.67	na	na	na	19°19′46.91″N	88° 0′48.94″W
Chetumal_A	Chetumal	Che	Agricultural	3.425	0.031	8.00	na	na	na	18°28′47.08″N	88°37′33.51″W
Tizimin_C	Tizimin	Tiz	City	3.511	0.046	7.27	na	na	na	21° 8′54.59″N	88° 9′41.44″W
Tizimin_N	Tizimin	Tiz	Natural	3.505	0.042	3.20	na	na	na	21°20′32.12″N	87°44′56.35″W
Tizimin_A	Tizimin	Tiz	Agricultural	3.484	0.033	17.33	na	na	na	21° 8′59.10″N	88° 3′51.14″W
Campeche_C	Campeche	Cam	City	3.383	0.022	7.44	na	na	na	19°49′26.01″N	90°33′30.94″W
Campeche_N	Campeche	Cam	Natural	3.458	0.026	18.00	na	na	na	19°53′34.55″N	90°20′59.28″W
Campeche_A	Campeche	Cam	Agricultural	3.444	0.030	20.33	na	na	na	19°45′35.82″N	90°28′56.18″W
Oxcutzcab_C	Oxcutzcab	Oxk	City	3.376	0.047	25.60	na	na	na	20°18′29.59″N	89°24′50.94″W
Oxcutzcab_N	Oxcutzcab	Oxk	Natural	3.599	0.031	3.08	na	na	na	20°14′59.08″N	89°32′33.09″W
Oxcutzcab_A	Oxcutzcab	Oxk	Agricultural	3.496	0.041	7.75	na	na	na	20°15′31.37″N	89°29′18.41″W
PlayaCarmen_A	PlayaCarmen	Pla	Agricultural	3.463	0.039	11.67	na	na	na	20°36′14.95″N	87° 7′37.31″W
PlayaCarmen_I	PlayaCarmen	Pla	Island	3.376	0.048	13.00	na	na	na	20°26′7.86″N	86°59′22.82″W
Chiquila_A	Chiquila	Chi	Agricultural	3.516	0.044	23.33	na	na	na	21°24′29.02″N	87°20′22.00″W
Merida_N	Merida	Mer	Natural	3.483	0.032	3.20	3.560	0.039	3.07	20°45′6.02″N	89°39′55.81″W
Merida_C	Merida	Mer	City	na	na	na	3.489	0.031	14.00	20°58′30.05″N	89°39′23.73″W
CiudadCarmen_N	CiudadCarmen	Ciu	Natural	3.538	0.039	8.17	3.579	0.026	8.83	18°29′6.85″N	91° 7′34.09″W
CiudadCarmen_A	CiudadCarmen	Ciu	Agricultural	3.466	0.043	13.50	3.552	0.036	9.50	18°46′54.65″N	90°56′25.27″W
CiudadCarmen_I	CiudadCarmen	Ciu	Island	na	na	na	3.539	0.041	5.60	18°39′46.82″N	91°46′12.47″W

C city, *A* agricultural, *I* Island, *N* natural. The region code is made of the first three letters of each region and is used to indicated regions in Fig. 3

### Body size measurements

To investigate variation in body size among different habitats, we measured intertegular distance (ITD) as the span between the two insertion points of the wings (tegulae) of each male. ITD is a good indicator of body size as it correlates well with dry body mass [104]. As body size is strongly correlated with species mobility [27], it is also considered a good indicator of dispersal ability. The coefficient of variation (CV = SD/mean) of ITD was also calculated and compared.

Measurements were performed using a stereo microscope (Olympus SZX7) with an integrated camera to record pictures and the digital measurement tool in the cellSens software v1.6. Of the originally sampled individuals, we randomly chose and measured 20 individuals from each site per bee species, totalling 440 male *E. dilemma* and 120 male *E. viridissima*.

### Land use variables

Habitats differ in landscape characteristics and environmental conditions, such as road density and land use, which may all be associated with shifts in body size and which may affect species at different spatial scales [21, 29]. To account for the scale dependency of body size-environmental relationships [105], we quantified *road density*, a metric for fragmentation, at 6 spatial scales (250 m, 500 m 750 m 1000 m, 2000 m and 3000 m) using Quantum GIS (QGIS.org, 2020) with data obtained from Geofabrik GmbH. We did so by computing the total length of roads contained in a circle centred on the coordinates of each site and at the six different radii corresponding to the 6 spatial scales. We chose to include three relatively wide spatial scales (1000 m, 2000 m and 3000 m) as both *E. dilemma* and *E. viridissima* are relatively big (mean ITD 3.47 mm and 3.54 mm respectively, see results), slightly bigger than the average European honey bee (mean ITD 3.3 mm; [27]). They are also known for their great flight capabilities (potentially up to 40 km, [67]). Using Google Earth Pro (Google Earth Version 7.3.3), we characterized land use cover by quantifying at the same six spatial scales the proportions of six different land use types that might be relevant for resource acquisition: forest (*Forest*), agricultural areas (*Agriculture*), semi-natural areas (*SeminaturalAreas*), impervious surface (*ImperviousSurface*), urban green spaces (*UrbanGreenSpace*) and water (*Water*) (Additional file 4: Table S4)*.*

### Statistical analyses

To investigate whether the body size of individuals from urban areas, agricultural areas, natural areas and islands differed among each other, we used linear mixed models (LMMs, lmer function of the ‘lme4’ package [106]), with habitat type, species and their interaction as fixed factors and sample site as a random effect factor. We then assessed the significance of effects using the Wald chi-square test (type II). We performed the same analysis for each species separately and tested for the significance of differences in body size between habitat types with Tukey’s HSD post hoc method using the R package ‘multcomp’ [107]. Using linear models (lm function in base R), we also tested for differences in body size variation (CV of ITD) among habitat types, both for *E. dilemma* and for *E. viridissima*.

To assess how the four habitat types (natural, agricultural, city and island) are characterised in terms of land use variables that we quantified, we ran a series of Kruskal–Wallis tests followed by Dunn tests for multiple comparisons to test for differences in land use variables among habitat types. We compared proportions (not normally distributed, hence our use of the non-parametric Kruskal–Wallis test) of land use characterised at the 1000 m scale because this was the scale at which sites were assigned to the three mainland habitat types [88].

To investigate the land use variables which best explained the observed variability in body size, we ran LMMs using the lmer function of the R package ‘lme4’ [106], with land uses at the scale at which the absolute value of their correlation coefficient with body size was highest. For *E. dilemma*, scales were: 3000 m for *Agriculture*, 2000 m for *Forest*, 3000 m for *ImperviousSurface*, 1000 m for *RoadDensity*, 2000 m for *SeminaturalAreas*, 500 m for *UrbanGreenSpace* and 2000 m for *Water* (Additional file 5: Table S5). Sample site was added as a random factor. As insufficient data were available to run the same analysis for *E. viridissima*, we undertook this analysis only for *E. dilemma*. Values for all predictors were standardized (transformed to z-scores) before running each model. Model assumptions were checked visually and were found to conform to expectations (residuals normally distributed, homogeneity of variance, linearity). The presence of outliers was checked using Cook’s distance within the R package ‘car’ [108]. The variance inflation factor (vif threshold = 5) was used to detect collinearity. As high collinearity was detected for *RoadDensity* (vif = 29.5) in the initial global model for *E. dilemma*, we ran subsequent analyses without *RoadDensity*. The function *Moran.I* from the package ‘ape’ [109] was used to detect spatial autocorrelation; none was found.

All statistical analyses were performed using the statistical software R v. 4.0.4.

## Supplementary Information

**Additional file 1: Table S1.** Results Pairwise (between habitats) comparisons of body size (ITD)

**Additional file 2: Table S2.** Comparison of land use variables across habitats

**Additional file 3: Table S3.** Results from Linear Mixed Model fitting body size as response variable of land use variables

**Additional file 4: Table S4.** Site characteristics

**Additional file 5: Table S5.** Correlation coefficients between body size (ITD) of *Euglossa dilemma* and land uses at different scales

**Additional file 6: Table S6.** Body Size measurements

**Additional file 7: Figure S1.** Mean estimates and confidence intervals (95%) of body size (measured as intertegular distance, ITD) for *Euglossa dilemma* across the four habitats ‘Agricultural’, ‘City’, ‘Island’ and ‘Natural’. Different letters indicate statistically significant differences (p < 0.05).

**Additional file 8: Figure S2.** Mean estimates and confidence intervals (95%) of body size (measured as intertegular distance, ITD) for *Euglossa viridissima* across the four habitats ‘Agricultural’, ‘City’, ‘Island’ and ‘Natural’. Different letters indicate statistically significant differences (p < 0.05).

## Data Availability

The data generated and analysed for this study are available as additional files.

## Abbreviations

CV: Coefficient of variation
ITD: Intertegular distance
SD: Standard deviation
