# Supplementary figures and images for: Anthropogenic effects on the body size of two neotropical orchid bees
Source: BMC Ecol Evol. 2022 Aug 2;22:94. doi: 10.1186/s12862-022-02048-z (PMC9347145; doi:10.1186/s12862-022-02048-z)

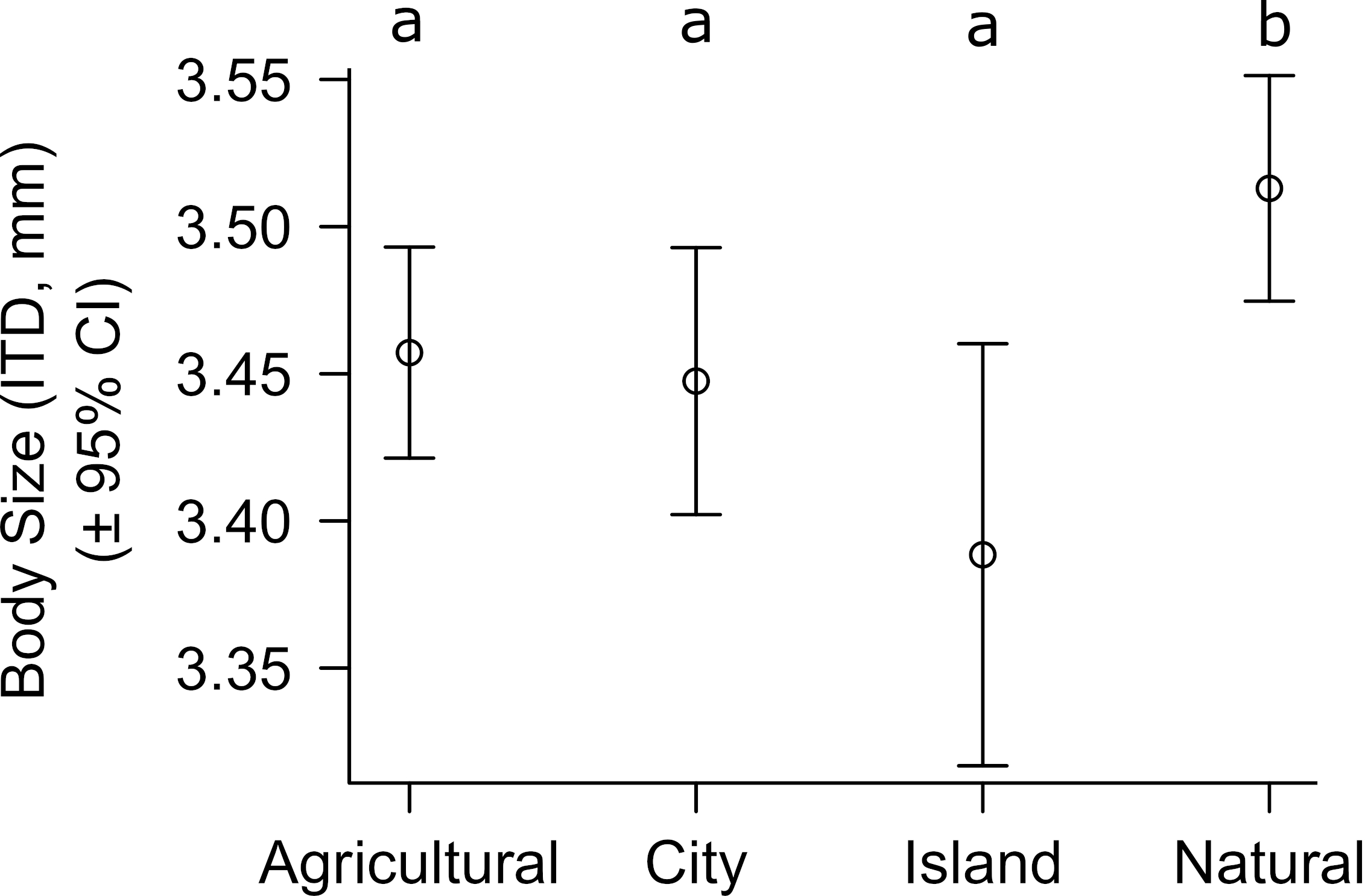

Supplement: Supplementary file 7 — Additional file 7: Figure S1. Mean estimates and confidence intervals (95%) of body size (measured as intertegular distance, ITD) for Euglossa dilemma across the four habitats ‘Agricultural’, ‘City’, ‘Island’ and ‘Natural’. Different letters indicate statistically significant differences (p < 0.05). [file 12862_2022_2048_MOESM7_ESM.jpeg]

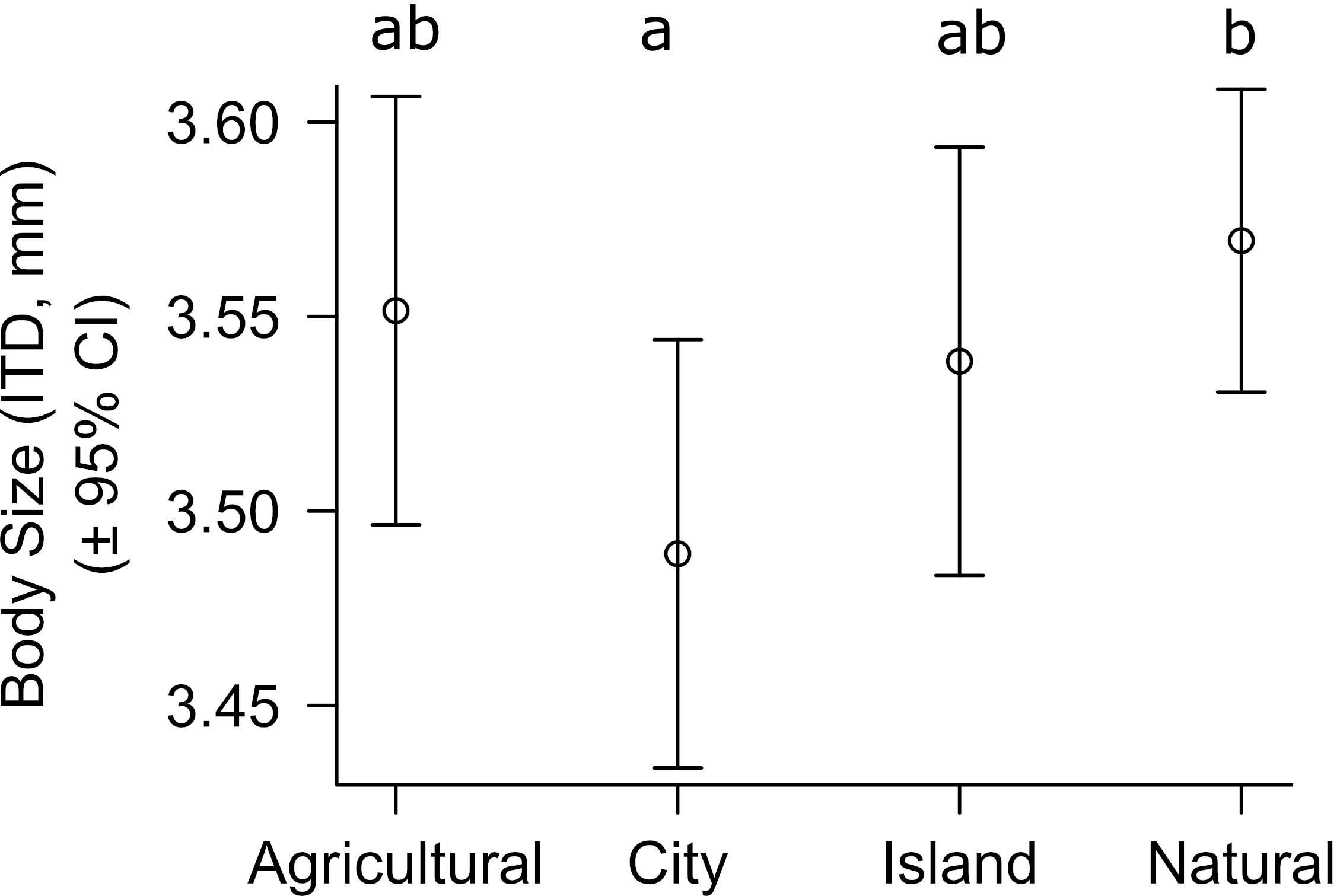

Supplement: Supplementary file 8 — Additional file 8: Figure S2. Mean estimates and confidence intervals (95%) of body size (measured as intertegular distance, ITD) for Euglossa viridissima across the four habitats ‘Agricultural’, ‘City’, ‘Island’ and ‘Natural’. Different letters indicate statistically significant differences (p < 0.05). [file 12862_2022_2048_MOESM8_ESM.jpeg]
